# MXene/Carbon Nanotube/Poly(ethylene oxide) Heterostructured Interface for Polysulfide Regulation in Lithium–Sulfur Batteries

**DOI:** 10.3390/molecules31173078

**Published:** 2026-09-01

**Authors:** Bingjie Liu, Linin Wang, Yangchuan Ke

**Affiliations:** College of Science, China University of Petroleum, Beijing 102200, China; liubingjiesta@163.com (B.L.); neauwll@163.com (L.W.)

**Keywords:** MXene, carbon nanotubes, poly(ethylene oxide), lithium–sulfur batteries, polysulfide regulation, separator, Li_2_S deposition, heterostructured interface

## Abstract

Lithium–sulfur (Li–S) batteries are promising next-generation energy-storage systems but are severely hindered by polysulfide shuttling, sluggish sulfur redox kinetics, and unstable Li_2_S nucleation/growth behavior. Herein, a multifunctional MXene/carbon nanotube/poly(ethylene oxide) (MX/CNT/PEO)-modified separator is developed to regulate polysulfide behavior and improve interfacial electrochemical stability. In this composite architecture, MXene provides polar sites for lithium polysulfide adsorption, while carbon nanotubes construct interconnected conductive networks and suppress MXene restacking. The incorporation of poly(ethylene oxide) improves interfacial continuity and introduces additional oxygen-containing functionalities within the composite framework. Benefiting from the integrated effects of polar adsorption, conductive pathways, and structural integration, the MX/CNT/PEO-modified separator effectively suppresses polysulfide diffusion, reduces charge-transfer resistance, and promotes more favorable Li_2_S nucleation/growth behavior. Electrochemical analysis shows that the charge-transfer resistance decreases from 175.9 Ω for pristine PP to 15.7 Ω for the MX/CNT/PEO@PP separator, corresponding to a 91.1% reduction. Potentiostatic Li_2_S deposition analysis further supports favorable Li_2_S nucleation/growth behavior on the MX/CNT/PEO-modified interface, after background subtraction. As a result, the Li–S cell with the MX/CNT/PEO@PP separator delivers a high initial discharge capacity of 1613 mAh g^−1^ at 0.1 C and maintains average capacities of 1331.1 and 803.1 mAh g^−1^ at 0.1 and 2 C during rate testing, respectively. During long-term cycling at 2 C, the cell retains 319.3 mAh g^−1^ after 1000 cycles, with an average capacity decay rate of 0.064% per cycle. This work provides a rational composite-interlayer design strategy for multifunctional separator materials in high-performance Li–S batteries.

## 1. Introduction

Lithium–sulfur (Li–S) batteries have attracted extensive attention as promising next-generation energy-storage systems owing to their ultrahigh theoretical specific capacity of 1675 mAh g^−1^, high gravimetric energy density of 2600 Wh kg^−1^, and the abundance and environmental benignity of sulfur [1,2,3]. Despite these advantages, the practical implementation of Li–S batteries remains severely hindered by the shuttle effect of soluble lithium polysulfides (Li_2_Sx, 4 ≤ x ≤ 8), sluggish sulfur redox kinetics, and uncontrolled Li_2_S deposition during cycling [4,5,6]. These issues lead to active-material loss, electrode passivation, low sulfur utilization, and rapid capacity fading, thereby limiting the long-term stability and rate capability of Li–S batteries [7,8,9,10,11,12]. Therefore, developing effective strategies to regulate polysulfide behavior and improve interfacial stability remains a critical prerequisite for achieving high-performance Li–S batteries.

To address these challenges, extensive efforts have been devoted to sulfur-host design, electrolyte optimization, and interfacial engineering strategies [13,14,15]. Among these approaches, separator engineering has emerged as an effective and practical strategy because it can directly regulate polysulfide migration without altering the cathode composition [16,17,18]. An ideal functional separator should not only suppress polysulfide diffusion but also provide effective polysulfide confinement, maintain interfacial stability, and support durable electrochemical reactions during prolonged cycling [19].

Carbon-based materials, including porous carbon, graphene, carbon nanofibers, and carbon nanotubes (CNTs), have been widely investigated as separator modification materials owing to their excellent electrical conductivity, structural tunability, and chemical stability [20,21,22]. In particular, CNTs can construct interconnected conductive pathways and act as structural spacers to alleviate material aggregation and maintain efficient electron transport during cycling [23]. However, the intrinsically nonpolar nature of carbon materials results in limited chemical affinity toward lithium polysulfides, making it difficult to achieve strong polysulfide confinement and stable electrochemical interfaces simultaneously [24,25].

To further enhance polysulfide confinement and interfacial stability, various polar materials, including metal oxides, sulfides, nitrides, phosphides, and two-dimensional (2D) materials, have been introduced into Li–S systems [26,27,28]. Among these candidates, MXenes have attracted considerable attention owing to their metallic conductivity, abundant surface terminations, and strong chemical affinity toward lithium polysulfides [29,30,31]. The polar surface functionalities of MXenes enable effective polysulfide adsorption and strong interactions with lithium polysulfides, thereby improving polysulfide retention and interfacial stability [32,33]. Nevertheless, MXene nanosheets tend to undergo restacking during fabrication and cycling, which reduces the accessibility of active sites and hinders efficient electron transport within the separator framework [34]. Therefore, relying solely on MXene adsorption remains insufficient for constructing a durable and efficient multifunctional separator in practical Li–S batteries.

In parallel, polymer-assisted strategies have emerged as promising approaches for improving interfacial compatibility and structural stability in Li–S systems [35,36]. Among various polymers, poly(ethylene oxide) (PEO) has attracted considerable attention because of its abundant ether oxygen groups and favorable compatibility with lithium-salt-based electrolytes [18,37]. Although bulk PEO generally suffers from limited ionic conductivity at room temperature, its incorporation into composite separator architectures can introduce additional oxygen-containing functionalities and improve electrolyte affinity within the separator framework [38,39,40]. Therefore, integrating polymer phases with conductive and polar components provides opportunities for constructing multifunctional composite separators capable of regulating polysulfide behavior and improving interfacial stability [41].

Beyond cathode design, separator engineering has been widely recognized as an effective and practical strategy for suppressing polysulfide migration without substantially altering the cathode structure [42,43,44]. Conventional polyolefin separators such as polypropylene (PP) possess chemically inert and hydrophobic surfaces, exhibiting weak affinity toward lithium polysulfides and limited capability for polysulfide retention [45,46]. Consequently, functional separator modification using conductive and polar interlayers has attracted increasing attention [47,48]. Ideally, a separator modification layer should simultaneously provide efficient polysulfide confinement, rapid electron transport, and stable electrochemical interfaces during prolonged cycling [49]. However, many reported interlayer strategies remain functionally separated, where conductivity enhancement and polysulfide adsorption are achieved independently rather than synergistically. Such functional decoupling limits the development of efficient and durable multifunctional separators for practical Li–S batteries.

Inspired by the above considerations, an MXene/carbon nanotube/poly(ethylene oxide)-based multifunctional separator was designed to address the functional decoupling commonly encountered in conventional interlayer strategies. In this composite architecture, MXene serves as a polar component for immobilizing lithium polysulfides [29,32]. Carbon nanotubes establish interconnected conductive pathways while acting as structural spacers to suppress MXene restacking and maintain efficient electron transport [23,25]. Meanwhile, the incorporation of PEO improves interfacial compatibility and introduces additional oxygen-containing functionalities within the composite framework [37,39]. As a result, the integrated separator forms a conductive–polar heterostructure capable of enhancing polysulfide confinement, improving interfacial stability, and promoting durable electrochemical performance during cycling [50].

In this work, an MXene/carbon nanotube/poly(ethylene oxide)-coated polypropylene separator (denoted as MX/CNT/PEO@PP) was fabricated and systematically investigated for lithium–sulfur batteries. The composite separator integrates polar MXene nanosheets, conductive CNT networks, and a polymer-assisted interfacial framework to construct a multifunctional heterostructure for polysulfide regulation. The structural characteristics, surface chemistry, and polysulfide adsorption behavior of the composite separator were comprehensively studied, while density functional theory (DFT) calculations were employed to elucidate the interfacial interaction mechanisms [51,52]. Electrochemical evaluations demonstrate that the MX/CNT/PEO@PP separator effectively suppresses polysulfide migration, reduces charge-transfer resistance, enhances rate capability, and contributes to more uniform Li_2_S deposition behavior [53,54]. The combined experimental and theoretical results reveal the important role of the MX/CNT/PEO heterostructure in strengthening polysulfide interactions and improving electrochemical stability. This work provides a rational heterostructure design strategy for developing multifunctional separator materials in high-performance lithium–sulfur batteries.

## 2. Results

### 2.1. Structural Design and Fabrication Mechanism

**Figure 1:**
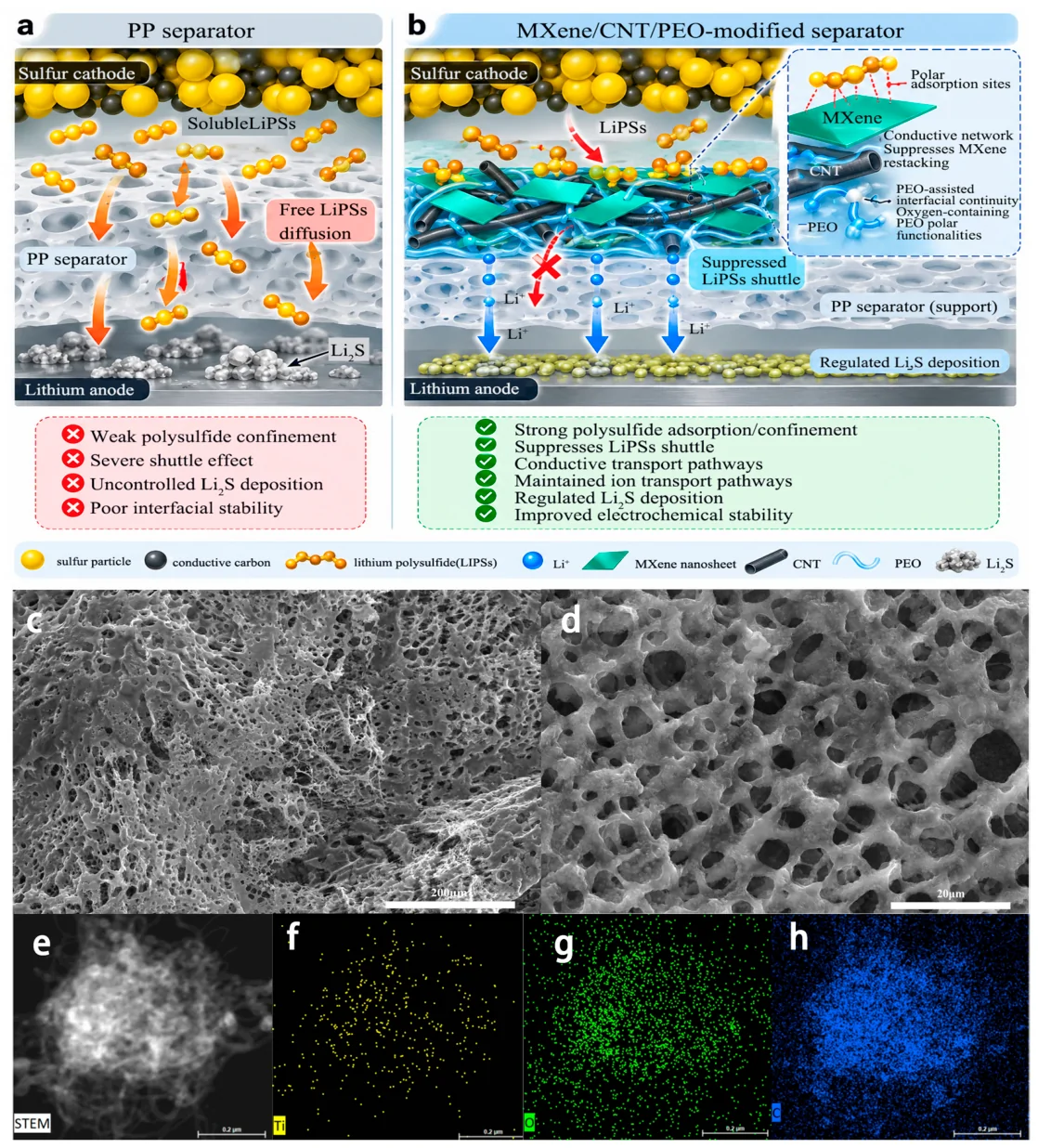
Design concept and structural characterization of the MX/CNT/PEO-modified separator. (**a**) Schematic illustration of polysulfide diffusion and uncontrolled Li_2_S deposition in a Li–S cell with a pristine PP separator. (**b**) Schematic illustration of the MX/CNT/PEO-modified separator, where the MX/CNT/PEO interlayer combines polar adsorption sites from MXene, conductive pathways from CNTs, and PEO-assisted interfacial continuity to suppress polysulfide shuttling and regulate Li_2_S nucleation/growth behavior. (**c**,**d**) Cross-sectional SEM images of the MX/CNT/PEO composite layer obtained after liquid-nitrogen-assisted brittle fracture, showing a porous and interconnected structure. (**e**) STEM image of the MX/CNT/PEO composite. (**f**–**h**) Elemental mapping images of Ti, O, and C, respectively, confirming the presence and distribution of MXene-related Ti species, oxygen-containing groups, and carbonaceous components in the composite.

Figure 1 illustrates the structural design concept and microstructural characteristics of the MX/CNT/PEO-modified separator. As schematically shown in Figure 1a, the pristine PP separator mainly acts as a porous physical barrier and shows limited capability for polysulfide confinement, resulting in free LiPS diffusion, severe shuttle effect, and uncontrolled Li_2_S deposition. In contrast, the MX/CNT/PEO-modified separator introduces a multifunctional interfacial layer on the PP substrate (Figure 1b). In this architecture, MXene nanosheets provide polar adsorption sites for LiPS immobilization, CNTs construct interconnected conductive pathways and suppress MXene restacking, while the incorporated PEO phase improves interfacial continuity and introduces oxygen-containing functionalities within the composite framework. Such an integrated structure is expected to combine polysulfide confinement, conductive transport, and maintained ion-accessible pathways, thereby contributing to regulated Li_2_S nucleation/growth behavior and improved interfacial stability.

Cross-sectional SEM images obtained after liquid-nitrogen-assisted brittle fracture (Figure 1c,d) reveal a well-developed porous and interconnected architecture throughout the composite layer. The hierarchical porous structure is beneficial for electrolyte infiltration and provides abundant interfacial contact sites for electrochemical reactions. No obvious structural collapse or severe aggregation is observed, indicating the successful formation of a continuous MX/CNT/PEO framework.

Furthermore, the STEM image (Figure 1e) and corresponding elemental mapping images (Figure 1f,h) demonstrate the distribution of Ti, O, and C elements throughout the composite region, respectively. The Ti signal is associated with MXene nanosheets, the C signal mainly corresponds to the CNT network and carbonaceous components, and the O signal is related to oxygen-containing surface groups and the PEO-containing phase. These results support the homogeneous integration of MXene, CNTs, and PEO within the composite separator framework. Additional SEM images at different magnifications (Figure S2, Supporting Information) further confirm the structural continuity and porous nature of the composite layer.

To further examine the structural characteristics of the composite, X-ray diffraction (XRD) and Raman spectroscopy analyses were conducted (Figure S1). As shown in Figure S1a, the characteristic low-angle (002) reflection of Ti_3_C_2_T_x_ MXene remains visible after composite formation, indicating that the layered MXene structure is partially preserved in the MX/CNT/PEO composite. The broad diffraction peak around 25–26° can be assigned to the graphitic (002) plane of CNTs, while the reflections around 19° and 23° are associated with crystalline PEO domains. These diffraction features indicate the coexistence of MXene, CNTs, and PEO within the composite framework. No dominant crystalline TiO_2_ phase is assigned solely from the XRD pattern; possible surface-oxidized Ti–O/TiO_2_-like species are further evaluated by XPS.

Raman spectroscopy was further performed to analyze the carbonaceous structure of the composite (Figure S1b). The Raman spectrum exhibits characteristic D and G bands at approximately 1350 and 1580 cm^−1^, respectively. The D band is associated with defective/disordered carbon, while the G band corresponds to sp^2^-hybridized graphitic carbon, confirming the presence of CNT-derived conductive carbon networks in the composite.

**Figure 2:**
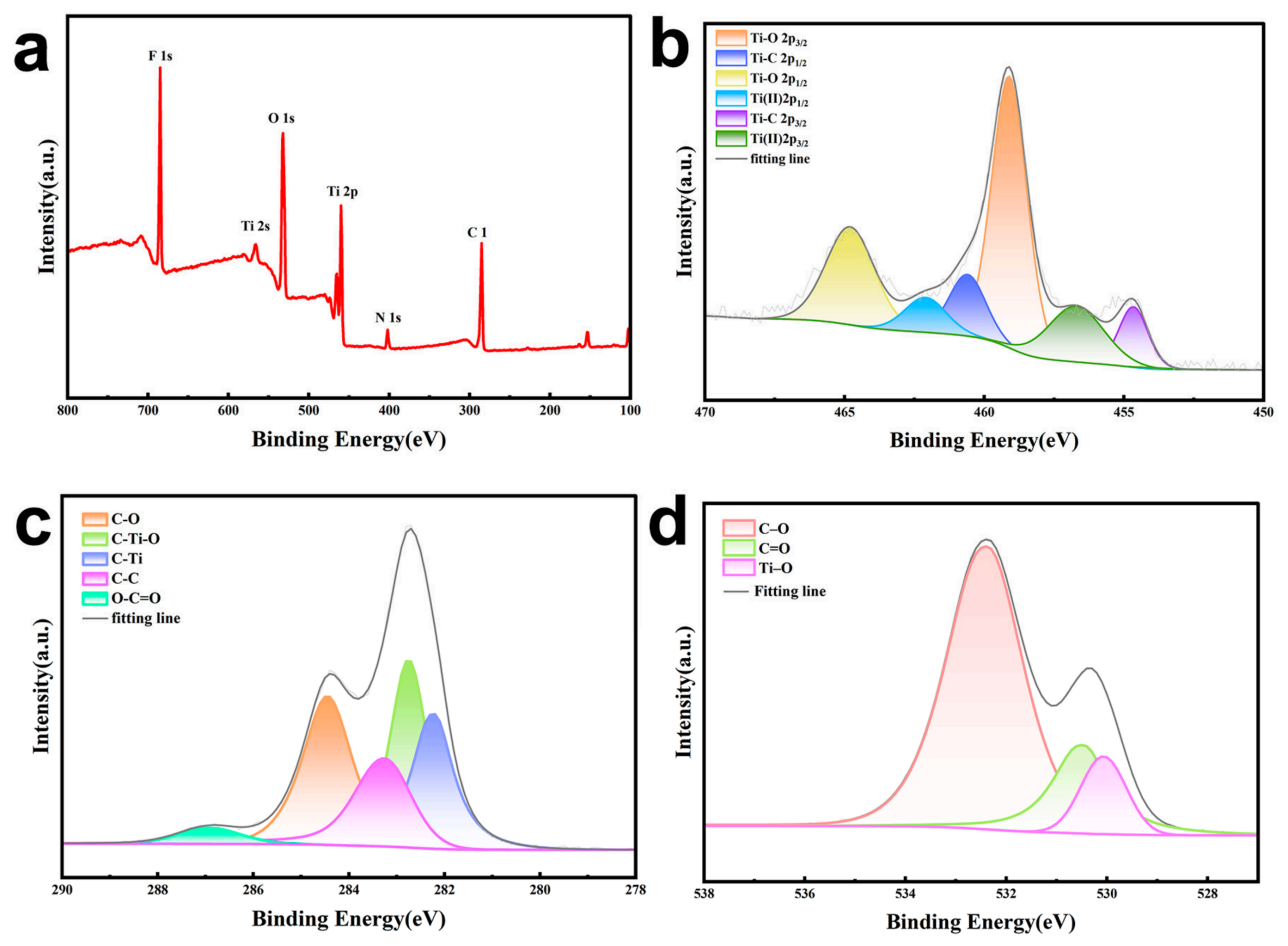
XPS analysis of the MX/CNT/PEO composite. (**a**) Survey spectrum confirming the presence of Ti, C, O and F elements. (**b**) High-resolution Ti 2p spectrum deconvoluted into Ti–C, Ti–O and Ti–F components. (**c**) High-resolution C 1s spectrum with peaks assigned to C–C, C–O, C–Ti and O–C=O bonds. (**d**) High-resolution O 1s spectrum showing contributions from Ti–O, C–O and C=O species.

Following the structural characterization, X-ray photoelectron spectroscopy (XPS) was employed to investigate the surface chemical states and interfacial chemistry of the MX/CNT/PEO composite (Figure 2). The survey spectrum (Figure 2a) confirms the presence of Ti, C, O, and F elements, consistent with the integration of Ti_3_C_2_T_x_ MXene, CNTs, and PEO within the composite framework. A weak N 1s signal is also observed, which may be associated with trace residual solvent or surface-adsorbed species. The high-resolution Ti 2p spectrum (Figure 2b) can be deconvoluted into Ti–C and Ti–O/Ti(IV)-related species, indicating that the carbide framework of MXene is largely preserved after composite formation while partial surface oxidation occurs. Such oxygen-containing surface terminations may provide polar interaction sites for lithium polysulfide species.

The C 1s spectrum (Figure 2c) exhibits multiple bonding configurations, including C–C/C=C, C–Ti, C–O, and O–C=O species. The presence of C–Ti bonds further supports the structural integrity of MXene, while the C–C signal originates primarily from the conductive CNT framework. Oxygen-containing carbon species are associated with both functionalized CNTs and the PEO phase, suggesting effective integration among the composite components.

As shown in Figure 2d, the O 1s spectrum can be resolved into Ti–O, C–O, and C=O/O–C=O contributions, confirming the abundance of oxygen-containing functional groups within the hybrid interface. The corresponding binding energies and peak assignments are summarized in Table S2 (Supporting Information). These oxygen-related species arise collectively from MXene surface terminations, functionalized CNTs, and the polymer phase, reflecting a chemically heterogeneous interfacial environment.

Overall, the XPS results reveal a polar-rich interfacial chemistry and integrated hybrid framework within the MX/CNT/PEO composite. Such surface characteristics are expected to influence lithium polysulfide interactions and interfacial electrochemical behavior. To gain deeper insight into the adsorption behavior and interfacial interactions between lithium polysulfides and the composite interface, density functional theory (DFT) calculations were subsequently performed.

### 2.2. DFT Analysis of Interfacial LiPS Adsorption

To gain insight into the interfacial interactions between lithium polysulfides (LiPSs) and the composite separator, density functional theory (DFT) models were constructed by incorporating representative CNT fragments and PEO segments into a Ti_3_C_2_T_x_-based interface rather than considering a single-component substrate. This model was designed to better represent the heterogeneous characteristics of the MX/CNT/PEO composite, where LiPS species may interact simultaneously with conductive, polar, and polymeric domains.

The calculated adsorption energies indicate strong interactions between the composite interface and LiPS species. Specifically, Li_2_S_6_ and Li_2_S_8_ exhibit adsorption energies of −7.359 and −8.331 eV, respectively, whereas the interaction with S_8_ is comparatively weaker (−5.693 eV). The stronger adsorption toward soluble LiPS intermediates suggests preferential stabilization of polysulfides relative to elemental sulfur, which is beneficial for suppressing polysulfide migration and retaining active sulfur species within the cathode region.

The strong adsorption behavior can be attributed to the coexistence of MXene surface terminations, oxygen-containing functional groups, and conductive CNT domains within the heterogeneous interface. This multifunctional framework provides abundant interaction sites for polysulfide adsorption and confinement.

These theoretical results indicate that the MX/CNT/PEO heterointerface provides a chemically favorable environment for immobilizing soluble polysulfides. The strong adsorption behavior is consistent with the experimentally observed polysulfide retention characteristics. To further verify the influence of the composite interface on polysulfide migration, polysulfide diffusion experiments were subsequently conducted.

**Figure 3:**
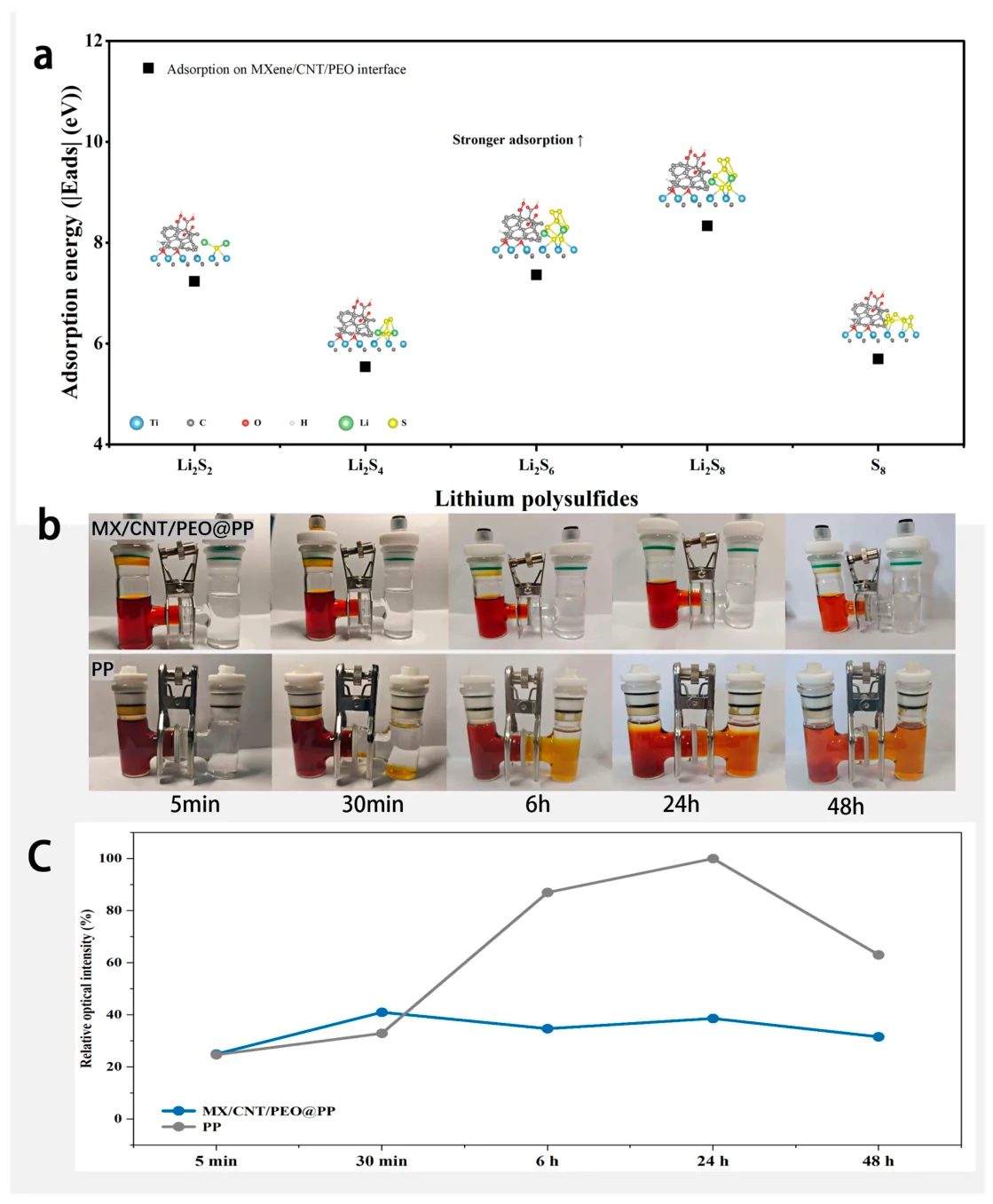
Interfacial polysulfide adsorption and diffusion-regulation behavior of the MX/CNT/PEO-modified separator. (**a**) Calculated adsorption energies of Li_2_S_2_, Li_2_S_4_, Li_2_S_6_, Li_2_S_8_, and S_8_ on the MXene/CNT/PEO heterointerface. The adsorption energies are presented as absolute values (|Eads|) for comparison, where a larger |Eads| indicates stronger adsorption. (**b**) Visual polysulfide diffusion tests using H-type cells separated by MX/CNT/PEO@PP and pristine PP separators at different diffusion times. The upper row corresponds to the MX/CNT/PEO@PP separator, and the lower row corresponds to the pristine PP separator. (**c**) Semi-quantitative optical-intensity analysis of the receiving chamber extracted from the diffusion photographs in panel (**b**), showing the suppressed polysulfide permeation of the MX/CNT/PEO@PP separator.

Density functional theory (DFT) calculations were performed to probe the interfacial interactions between lithium polysulfides (LiPSs) and the MX/CNT/PEO heterointerface. As shown in Figure 3a, the MX/CNT/PEO interface exhibits strong adsorption toward soluble polysulfide species, with calculated adsorption energies of −7.359 eV for Li_2_S_6_ and −8.331 eV for Li_2_S_8_, respectively. In contrast, the interaction with elemental sulfur (S_8_) is comparatively weaker (−5.693 eV). Such preferential adsorption toward soluble LiPS intermediates indicates strong affinity between the polar heterointerface and polysulfide species. This adsorption behavior may arise from the cooperative contributions of MXene surface terminations, oxygen-containing functionalities, and conductive CNT domains, which collectively provide multiple interaction sites within the composite framework.

The strong interfacial affinity predicted by DFT is further reflected in the polysulfide diffusion experiments (Figure 3b). Compared with the pristine PP separator, the MX/CNT/PEO-modified separator markedly suppresses polysulfide permeation, whereas rapid color diffusion is observed in the PP system. Notably, the receiving chamber of the MX/CNT/PEO@PP system remains much clearer even after prolonged diffusion, indicating substantially retarded migration of soluble LiPS species.

To provide semi-quantitative support for the visual diffusion results, the optical intensity of the receiving chamber was extracted from the diffusion photographs and normalized as a function of diffusion time (Figure 3c). The pristine PP separator shows a much faster increase in optical intensity, whereas the MX/CNT/PEO@PP separator maintains significantly lower intensity throughout the test, further confirming its suppressed polysulfide permeation. These results collectively suggest that the MX/CNT/PEO interface provides a chemically favorable environment for regulating polysulfide behavior.

**Figure 4:**
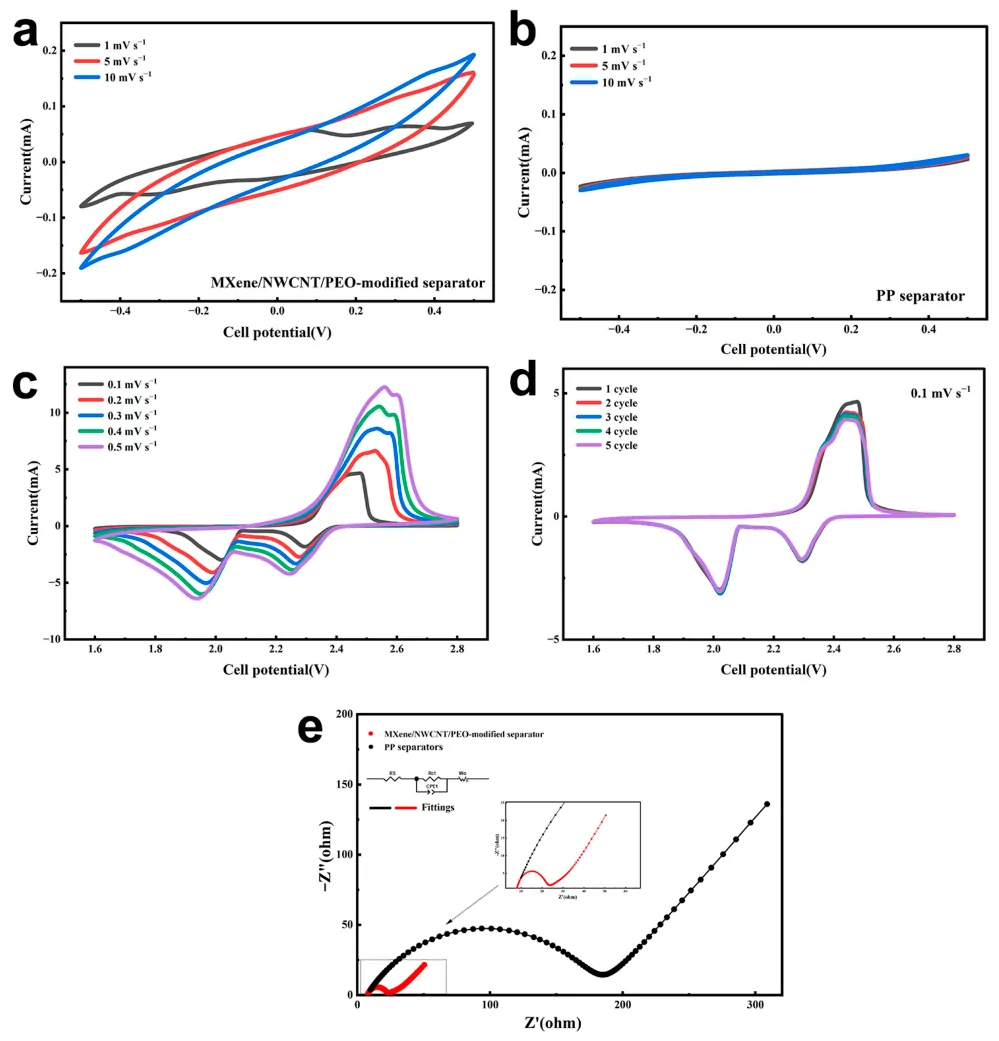
Polysulfide redox kinetics and interfacial charge-transfer characteristics. (**a**) CV curves of the MX/CNT/PEO-based Li_2_S_6_ symmetric cell at scan rates of 1, 5, and 10 mV s ^−1^. (**b**) CV curves of the pretreated carbon paper control symmetric cell under the same conditions. (**c**) CV curves of the Li–S cell with the MX/CNT/PEO@PP separator at scan rates from 0.1 to 0.5 mV s ^−1^. (**d**) CV profiles of the Li–S cell with the MX/CNT/PEO@PP separator during the initial five cycles at 0.1 mV s ^−1^. (**e**) EIS Nyquist plots of Li–S cells with pristine PP and MX/CNT/PEO@PP separators; the inset shows the high-frequency region, and the solid lines represent the fitted results.

To experimentally probe the redox behavior of lithium polysulfides (LiPSs), Li_2_S_6_ symmetric cells assembled with identical electrodes were employed. As shown in Figure 4a, the MX/CNT/PEO-containing composite electrode exhibited pronounced redox currents that increased with increasing scan rate, indicating a favorable interfacial response toward LiPS redox reactions. In contrast, the symmetric cell based on pretreated pristine carbon paper electrodes displayed much weaker capacitive-like responses under the same conditions (Figure 4b), suggesting limited LiPS redox activity of the carbon paper substrate. The enhanced faradaic response of the composite-coated electrode can be attributed to the integrated contribution of the conductive CNT network and polar MXene-containing interface, which provide more accessible electrochemically active sites for LiPS redox reactions. It should be noted that this comparison demonstrates the enhanced LiPS redox response of the MX/CNT/PEO-containing composite electrode relative to the pretreated carbon paper substrate, while mass-matched coating controls would be required to fully decouple the contribution of the composite material from that of the coating architecture.

The redox behavior of the Li–S full cell with the MX/CNT/PEO-modified separator was further evaluated by CV measurements. As shown in Figure 4c, the redox peak currents increase progressively with increasing scan rate while maintaining distinguishable peak features, indicating reversible sulfur redox behavior within the tested scan-rate range. Moreover, the CV curves collected during the initial five cycles at 0.1 mV s^−1^ (Figure 4d) show good overlap, suggesting stable electrochemical redox behavior of the MX/CNT/PEO@PP-based cell during the initial cycles. It should be noted that Figure 4c,d are used to evaluate the redox behavior and cycling reproducibility of the MX/CNT/PEO@PP-based cell itself, while the comparative kinetic improvement relative to pristine PP is mainly supported by the EIS, rate-performance, and cycling results.

Electrochemical impedance spectroscopy (EIS, Figure 4e) was further performed to evaluate the interfacial charge-transfer behavior of Li–S cells with different separators. The MX/CNT/PEO-modified separator exhibited a much smaller high-frequency semicircle than the pristine PP separator, indicating significantly reduced charge-transfer resistance (Rct). Based on the estimates from the Nyquist plots, the Rct was estimated to decrease from approximately 175.9 Ω for the pristine PP separator to 15.7 Ω for the MX/CNT/PEO-modified separator, corresponding to a reduction of about 91.1%. In addition, the steeper low-frequency region suggests more favorable ion diffusion and interfacial mass transport. These results indicate that the MX/CNT/PEO composite coating effectively improves interfacial charge-transfer characteristics and facilitates sulfur redox kinetics.

Overall, the modified separator serves not only as a physical barrier against polysulfide migration but also as a functional interfacial layer for polysulfide regulation. The strengthened LiPSs interactions and integrated conductive–polar architecture collectively contribute to improved interfacial electrochemical behavior, which supports the enhanced battery performance discussed below.

**Figure 5:**
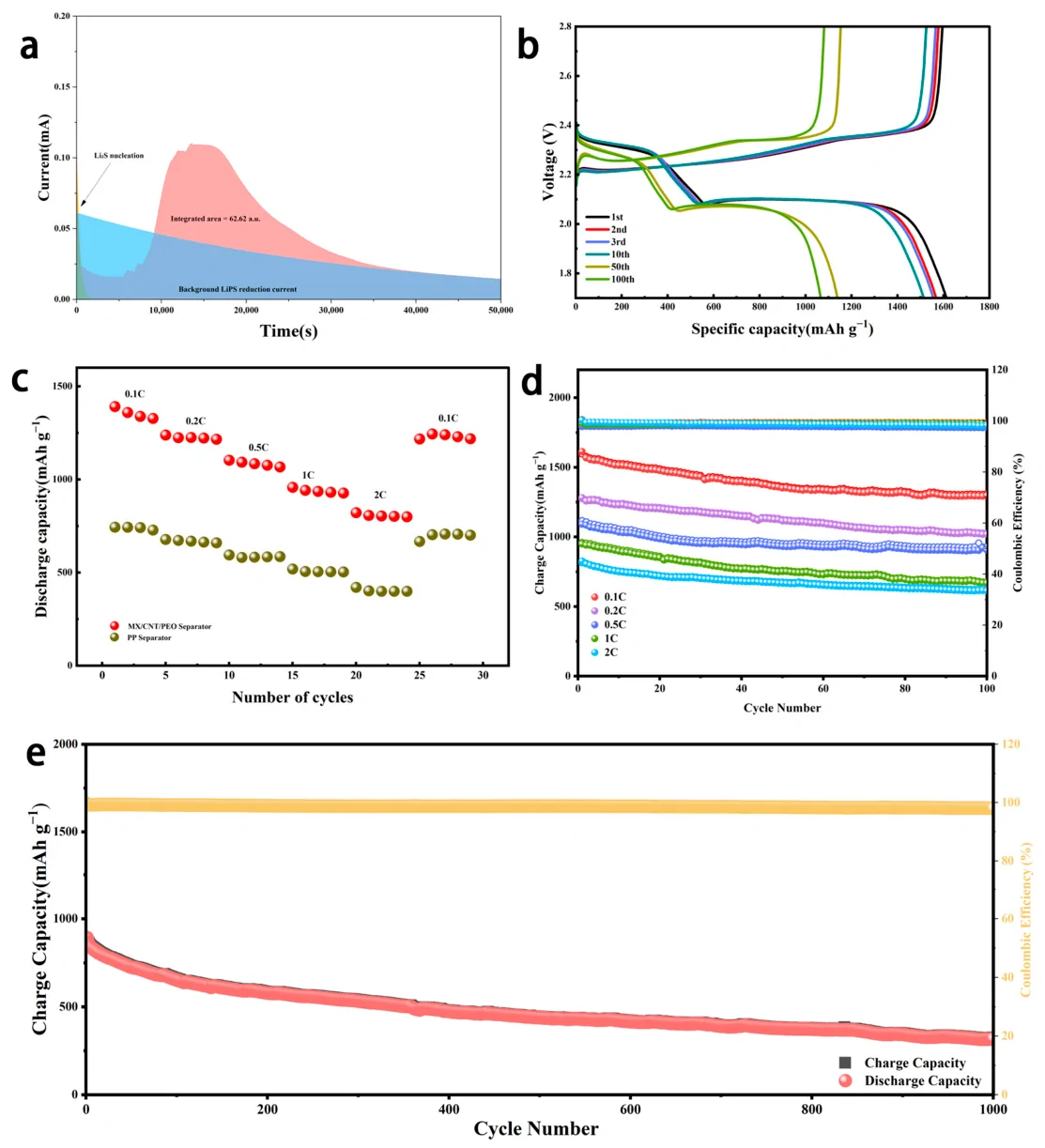
Electrochemical performance of Li–S cells with the MX/CNT/PEO-modified separator. (**a**) Li_2_S nucleation and growth behavior obtained from potentiostatic discharge, indicating facilitated nucleation kinetics. (**b**) Galvanostatic charge–discharge profiles at selected cycles, demonstrating stable voltage profiles. (**c**) Rate capability at current densities ranging from 0.1 to 2 C. (**d**) Cycling performance at different current densities. (**e**) Long-term cycling stability at 2 C over 1000 cycles with corresponding Coulombic efficiency, revealing excellent durability.

Potentiostatic Li_2_S deposition measurements were performed to probe the Li_2_S nucleation/growth behavior on the MX/CNT/PEO-modified interface (Figure 5a). The MX/CNT/PEO@PP cell exhibits a distinct Li_2_S nucleation current followed by a prolonged deposition region, indicating continuous Li_2_S nucleation and growth during the potentiostatic process. After background subtraction and integration of the Li_2_S nucleation/growth region, the integrated deposition area of the MX/CNT/PEO@PP cell was calculated to be 62.62 a.u. This result suggests that the MX/CNT/PEO-modified interface can provide active interfacial sites for Li_2_S nucleation and sustained growth. It should be noted that this analysis supports the Li_2_S nucleation/growth behavior of the modified separator, while direct comparison with pristine PP and post-deposition morphology would be required to further quantify Li_2_S deposition uniformity.

The galvanostatic charge–discharge profiles (Figure 5b) display well-defined voltage plateaus corresponding to typical sulfur redox reactions. The MX/CNT/PEO@PP cell delivered a high initial discharge capacity of 1613 mAh g^−1^ at 0.1 C, indicating high sulfur utilization during the initial activation process. It should be noted that this value corresponds to the first discharge cycle and was not used as the representative stabilized capacity for rate-performance evaluation.

The rate capability was further evaluated at 0.1, 0.2, 0.5, 1, and 2 C, corresponding to current densities of 167.5, 335, 837.5, 1675, and 3350 mA g^−1^, respectively (Figure 5c). During the rate test, the MX/CNT/PEO@PP cell delivered average discharge capacities of 1331.1, 1198.2, 1055.7, 938.7, and 803.1 mAh g^−1^ at 0.1, 0.2, 0.5, 1, and 2 C, respectively. In contrast, the pristine PP cell showed much lower average capacities of 727.1, 652.1, 570.8, 507.7, and 400.4 mAh g^−1^ at the same rates. When the current density returned to 0.1 C, the MX/CNT/PEO@PP cell recovered to an average capacity of 1229.9 mAh g^−1^, demonstrating good rate reversibility and improved interfacial stability.

The improved interfacial behavior is further reflected in cycling performance. As shown in Figure 5d, the MX/CNT/PEO@PP cell maintains stable capacity output under varying current densities, indicating good electrochemical reversibility during rate switching. More importantly, long-term cycling at 2 C (Figure 5e) demonstrates improved cycling stability over 1000 cycles. The MX/CNT/PEO@PP cell delivers an initial discharge capacity of 892.7 mAh g^−1^ at 2 C and retains 319.3 mAh g^−1^ after 1000 cycles, corresponding to a capacity retention of 35.8% and an average capacity decay rate of 0.064% per cycle. In contrast, the PP-based cell exhibits much faster capacity decay under the same conditions. The improved cycling stability is consistent with strengthened polysulfide confinement and more favorable Li_2_S nucleation/growth behavior at the MX/CNT/PEO-modified interface.

Overall, the MX/CNT/PEO interfacial layer effectively combines polysulfide confinement with improved interfacial electrochemical behavior. The integrated effects of polar polysulfide adsorption, conductive CNT-assisted electron pathways, and PEO-assisted interfacial continuity collectively contribute to improved rate capability and long-term cycling stability in Li–S batteries.

## 3. Discussion

The improved electrochemical performance of the MX/CNT/PEO@PP separator can be understood from the integrated effects of the conductive–polar composite coating. In conventional Li–S batteries, soluble lithium polysulfides readily migrate through the separator, resulting in active-material loss, parasitic reactions at the lithium anode, and rapid capacity decay. A functional separator is therefore expected to simultaneously restrict polysulfide migration, maintain efficient charge transfer, and provide a stable interfacial environment for sulfur redox reactions. In this work, the MX/CNT/PEO coating fulfills these functions through the cooperative contributions of MXene nanosheets, carbon nanotubes, and a low-content polymer phase.

MXene nanosheets play a dominant role in polysulfide immobilization owing to their polar surface terminations and strong affinity toward lithium polysulfides. The DFT adsorption calculations and polysulfide diffusion experiments indicate that the MXene-containing composite coating can effectively retard polysulfide migration. This result is consistent with previous studies showing that polar MXene surfaces can interact with lithium polysulfides more strongly than nonpolar carbon materials. However, MXene nanosheets alone tend to restack during slurry preparation and coating, which may reduce the accessibility of active sites and hinder mass/electron transport. The introduction of CNTs alleviates this limitation by acting as conductive spacers between MXene nanosheets. The interconnected CNT network improves electronic transport within the coating and helps maintain an open composite structure, thereby enhancing the utilization of polar adsorption sites.

The role of PEO should be interpreted carefully. In the present composite separator, PEO is not considered as an independent high-conductivity polymer electrolyte. Instead, the low-content PEO phase mainly functions as a polymer-assisted interfacial component. Its ether oxygen groups and oxygen-containing segments may provide additional polar environments for lithium-containing polysulfide species, while the polymer chains improve the continuity and integrity of the MXene/CNT coating. Such effects are beneficial for electrolyte affinity and interfacial contact within the separator coating. Therefore, the improvement brought by PEO is attributed primarily to interfacial compatibility, coating stability, and auxiliary polar interactions, rather than solely to enhanced lithium-ion conduction.

The electrochemical results further support the above structure–function relationship. The lower charge-transfer resistance of the MX/CNT/PEO@PP-based cell suggests more favorable interfacial charge transport compared with the pristine PP separator. Symmetric-cell CV measurements reveal enhanced redox response of lithium polysulfides, while Li_2_S deposition measurements indicate more sustained nucleation and growth behavior. These results suggest that the composite coating not only suppresses polysulfide migration but also provides a more favorable interface for sulfur redox conversion. Consequently, the cell with the MX/CNT/PEO@PP separator delivers improved rate capability and long-term cycling stability.

Compared with previously reported separator modification strategies, the present design integrates polar adsorption, conductive transport, and polymer-assisted interfacial stabilization into one composite coating. Carbon-based separators generally provide good electronic conductivity but limited chemical affinity toward polysulfides. MXene-based separators offer stronger polar adsorption but may suffer from nanosheet restacking. Polymer-containing separators can improve interfacial compatibility but often lack sufficient electronic conductivity. The MX/CNT/PEO composite coating combines these complementary advantages, thereby providing a balanced separator design for polysulfide regulation in Li–S batteries.

Nevertheless, several limitations remain. First, the present work mainly evaluates the separator under moderate sulfur loading conditions, and further studies under high sulfur loading and lean electrolyte conditions are required to assess its practical applicability. Second, although DFT adsorption calculations provide useful insight into polysulfide affinity, more advanced electronic-structure analyses and in situ characterizations would be valuable for clarifying the dynamic interfacial evolution during cycling. Third, the specific contribution of PEO could be further quantified through systematic polymer-content optimization, electrolyte uptake, wettability, ionic conductivity, and Li^+^ transference number measurements. Future work should therefore focus on optimizing the polymer content, improving the coating thickness and mass loading, and evaluating the separator in more practical cell configurations.

Overall, the MX/CNT/PEO@PP separator demonstrates that a polymer-assisted conductive–polar composite coating is an effective strategy for regulating polysulfide behavior and improving Li–S battery performance. The findings provide useful guidance for designing multifunctional separator coatings that combine chemical adsorption, conductive networks, and interfacial stabilization.

## 4. Materials and Methods

### 4.1. Synthesis of Ti_3_C_2_T_x_ MXene

Ti_3_C_2_T_x_ MXene nanosheets were synthesized through an in situ HF etching process using LiF and hydrochloric acid based on a modified minimally intensive layer delamination (MILD) protocol. Briefly, 3 g of LiF was dissolved in 60 mL of 9 M HCl under magnetic stirring. Subsequently, 3 g of Ti_3_AlC_2_ powder was gradually added to the solution, and the mixture was maintained at 45 °C under continuous stirring for 24 h to selectively etch the Al layer from Ti_3_AlC_2_.

After etching, the suspension was repeatedly washed with deionized water via centrifugation until the supernatant reached a near-neutral pH (~6). The resulting sediment was then redispersed in deionized water and subjected to mild sonication for 5 min, followed by centrifugation at 10,000 rpm to remove residual multilayer particles. Finally, the supernatant containing delaminated Ti_3_C_2_T_x_ nanosheets was collected for subsequent use.

### 4.2. Preparation of MXene/CNT Composite Suspension

Carboxylated multi-walled carbon nanotubes (MWCNTs) were first dispersed in deionized water at a concentration of 10 mg mL^−1^ and ultrasonicated for 60 min to obtain a homogeneous CNT suspension. The as-prepared Ti_3_C_2_T_x_ MXene dispersion had a concentration of 5 mg mL^−1^. To prepare the MXene/CNT composite suspension, the MXene dispersion and CNT suspension were mixed at a volume ratio of 10:1, corresponding to an MXene-to-CNT mass ratio of 5:1. For example, 3.03 mL of MXene dispersion and 0.303 mL of CNT suspension contained 15.15 mg of MXene and 3.03 mg of CNTs, respectively.

The resulting mixture was magnetically stirred for 24 h and further ultrasonicated for 120 min to promote homogeneous dispersion and interfacial contact between MXene nanosheets and CNTs. A stable MXene/CNT composite suspension was obtained for subsequent processing. The MXene:CNT mass ratio of 5:1 was selected based on preliminary dispersion behavior and coating-forming stability rather than systematic electrochemical optimization. At this ratio, MXene served as the dominant polar component for polysulfide interaction, while CNTs provided conductive pathways and helped suppress MXene restacking.

### 4.3. Preparation of MX/CNT/PEO Composite

Poly(ethylene oxide) (PEO, Mw = 600,000) was incorporated into the MXene/CNT composite system as a polymer-assisted interfacial component. Briefly, PEO was heated at 70 °C until it became fully softened/melted and was then added to the MXene/CNT composite suspension while still warm. The PEO content was controlled at 10 wt.% relative to the total mass of MXene and CNTs. Accordingly, when the designed total solid mass of the MX/CNT/PEO composite was 20 mg, the masses of MXene, CNTs, and PEO were 15.15, 3.03, and 1.82 mg, respectively.

The resulting mixture was vigorously shaken and magnetically stirred for 1 h, followed by ultrasonication for 30 min to promote homogeneous dispersion. Subsequently, the suspension was frozen and freeze-dried for 48 h to obtain the MX/CNT/PEO composite powder for separator fabrication. The PEO content of 10 wt.% was selected based on preliminary slurry dispersion and coating-forming stability rather than systematic electrochemical optimization.

### 4.4. Fabrication of MX/CNT/PEO@PP Separator

The as-prepared MX/CNT/PEO composite was mixed with acetylene black and poly (vinylidene fluoride) (PVDF) binder at a mass ratio of 8:1:1. N-methyl-2-pyrrolidone (NMP) was added as the solvent, and the mixture was ball-milled for 3 h to obtain a homogeneous slurry. The resulting slurry was uniformly coated onto one side of a commercial polypropylene (PP) separator via a doctor-blade coating process. The coated separator was subsequently dried under vacuum at 60 °C for 12 h to remove residual solvent. The obtained modified separator was denoted as MX/CNT/PEO@PP. The average mass loading of the coating layer was controlled at approximately 0.5 mg cm^−2^.

### 4.5. Cell Assembly and Electrochemical Measurements

CR2032-type coin cells were assembled in an argon-filled glovebox with oxygen and moisture levels below 0.1 ppm. The sulfur cathode was prepared by mixing the S/C composite, Super P, and PVDF binder at a mass ratio of 8:1:1 in NMP to form a homogeneous slurry. The slurry was coated onto aluminum foil and dried under vacuum at 60 °C for 12 h before use. The sulfur cathodes were punched into circular disks with a diameter of 12 mm, corresponding to an electrode area of 1.13 cm^2^. The sulfur loading of the cathode was controlled at approximately 2.0 mg cm^−2^, corresponding to approximately 2.26 mg of sulfur in each cathode. The sulfur cathode, lithium metal anode, and separator were sequentially stacked to assemble Li–S cells.

The electrolyte consisted of 1 M lithium bis(trifluoromethanesulfonyl)imide (LiTFSI) dissolved in a 1,3-dioxolane/1,2-dimethoxyethane (DOL/DME, *v*/*v* = 1:1) solvent containing 1 wt% LiNO_3_ additive. A fixed electrolyte volume of 20 μL was added to each cell, corresponding to an E/S ratio of approximately 8.8 μL mg^−1^.

Galvanostatic charge–discharge tests were performed using a LAND battery testing system within a voltage window of 1.7–2.8 V (vs. Li/Li^+^). The rate performance was evaluated at 0.1, 0.2, 0.5, 1, and 2 C, corresponding to current densities of 167.5, 335, 837.5, 1675, and 3350 mA g^−1^, respectively, based on the theoretical capacity of sulfur. Full-cell cyclic voltammetry (CV) was conducted within 1.6–2.8 V at scan rates of 0.1–0.5 mV s^−1^. Electrochemical impedance spectroscopy (EIS) was performed over a frequency range of 100 kHz to 0.01 Hz with an AC amplitude of 5 mV using a Biologic VMP3 electrochemical workstation.

### 4.6. Symmetric Cell Configuration for LiPSs Electrochemistry

To investigate the electrochemical conversion behavior of lithium polysulfides (LiPSs), Li_2_S_6_ symmetric cells were assembled using identical electrodes. The electrode slurry was prepared by mixing the MX/CNT/PEO composite, acetylene black, and poly(vinylidene fluoride) (PVDF) binder in N-methyl-2-pyrrolidone (NMP) at a mass ratio of 8:1:1. The resulting slurry was coated onto circular carbon paper current collectors with a diameter of 12 mm and dried under vacuum at 60 °C for 6 h. The mass loading of the composite material on each carbon paper electrode was approximately 2.5 mg, corresponding to an areal loading of 2.21 mg cm^−2^.

Two identical MX/CNT/PEO-coated carbon paper electrodes were assembled face-to-face using a polypropylene (PP) separator to construct the Li_2_S_6_ symmetric cells. A total of 50 μL of 0.3 M Li_2_S_6_ solution prepared in DOL/DME (*v*/*v* = 1:1) containing 1 M LiTFSI and 1 wt% LiNO_3_ was used as both the electrolyte and active redox medium.

Cyclic voltammetry measurements were carried out using a Biologic VMP3 electrochemical workstation within a potential window of −1.0 to 1.0 V at scan rates of 1, 5, and 10 mV s^−1^. Symmetric cells assembled with pretreated pristine carbon paper electrodes under the same conditions were used as control samples to evaluate the background LiPS redox response of the carbon paper substrate. The pristine carbon paper control electrodes were subjected to the same cutting and drying procedures before cell assembly.

### 4.7. DFT Computational Details

Density functional theory (DFT) calculations were performed using the Vienna Ab initio Simulation Package (VASP). The exchange–correlation interactions were described within the generalized gradient approximation (GGA) using the Perdew–Burke–Ernzerhof (PBE) functional, while the electron–ion interactions were treated using the projector augmented wave (PAW) method. A plane-wave cutoff energy of 400 eV was employed, and a 2 × 2 × 1 Monkhorst–Pack k-point mesh was used considering the relatively large surface supercell. The convergence criteria for electronic self-consistent calculations and ionic relaxation were set to 1 × 10^−4^ eV and 0.02 eV Å^−1^, respectively. Van der Waals interactions were considered using the DFT-D3 correction method. A vacuum layer of more than 15 Å was introduced along the z direction to avoid interactions between periodic images. Structural models were visualized using VESTA software (version 3).

Li_2_S_2_, Li_2_S_4_, Li_2_S_6_, Li_2_S_8_, and S_8_ were selected as representative sulfur species to evaluate their adsorption behavior on the MXene-based composite interface. The Ti_3_C_2_T_x_ MXene slab was used as the main polar adsorption surface because MXene is considered the dominant component responsible for LiPS affinity in the MX/CNT/PEO coating. Several possible adsorption configurations were considered for each sulfur species, and the most stable adsorption configuration was selected for adsorption-energy comparison.

The adsorption energy (E*_ads_*) was calculated according to the following equation:

$$E_{ads}=E_{surface+LiPSs}-E_{surface}-E_{LiPSs}$$

where E_(*sub+LiPSs*)_, E*_su_*_b_, and E*_LiPSs_* represent the total energies of the adsorption system, the clean substrate, and the isolated sulfur species, respectively. According to this definition, a more negative Eads indicates stronger adsorption. For clearer comparison, the absolute values of adsorption energies, |E*_ads_*|, are shown in Figure 3.

## 5. Conclusions

In summary, a multifunctional MX/CNT/PEO-modified separator was developed for regulating polysulfide behavior and improving interfacial electrochemical stability in Li–S batteries. In the composite interlayer, MXene nanosheets provide polar adsorption sites for LiPS immobilization, CNTs construct interconnected conductive pathways and suppress MXene restacking, while the low-content PEO phase improves interfacial continuity and contributes oxygen-containing functionalities within the composite framework. The integrated MX/CNT/PEO architecture effectively suppresses polysulfide diffusion, enhances interfacial charge-transfer behavior, and promotes more favorable Li_2_S nucleation/growth.

DFT calculations and polysulfide diffusion analysis support the strong affinity of the MXene-based polar interface toward sulfur species. Electrochemical analysis further shows that the MX/CNT/PEO@PP separator significantly reduces the charge-transfer resistance from 175.9 Ω for pristine PP to 15.7 Ω, corresponding to a 91.1% decrease. Potentiostatic Li_2_S deposition analysis further supports favorable Li_2_S nucleation/growth behavior on the MX/CNT/PEO-modified interface. Benefiting from these improved interfacial characteristics, the MX/CNT/PEO@PP cell delivers a high initial discharge capacity of 1613 mAh g^−1^ at 0.1 C and maintains average capacities of 1331.1 and 803.1 mAh g^−1^ at 0.1 and 2 C during rate testing, respectively. During long-term cycling at 2 C, the cell retains 319.3 mAh g^−1^ after 1000 cycles, with an average capacity decay rate of 0.064% per cycle.

Although the present study demonstrates the effectiveness of the full MX/CNT/PEO composite separator compared with pristine PP, the individual contributions of MXene, CNTs, and PEO have not been fully decoupled. Future work should include systematic component-control separators, PEO-content optimization, ion-transport measurements, and evaluation under more practical conditions such as higher sulfur loading, lean electrolyte, controlled separator coating thickness/loading, and scalable separator fabrication. This work provides a rational composite-interlayer design strategy for developing multifunctional separators in high-performance Li–S batteries.

## Data Availability

The data supporting the findings of this study are available from the corresponding author upon reasonable request.

## Supplementary Materials

The following supporting information can be downloaded at: https://www.mdpi.com/article/10.3390/molecules31173078/s1, Table S1: Adsorption energies of LiPS species on the MX/CNT/PEO interface; Figure S1: Additional structural characterization of the MX/CNT/PEO composite; Figure S2: Additional SEM images of the MX/CNT/PEO composite at different magnifications; Table S2: Binding energies and assignments of XPS peaks for the MX/CNT/PEO composite. The references cited in the Supplementary Materials, including those related to DFT calculations and structural visualization, are included in the main reference list [55,56,57,58,59,60].
