# Supplementary material for: MXene/Carbon Nanotube/Poly(ethylene oxide) Heterostructured Interface for Polysulfide Regulation in Lithium–Sulfur Batteries
Source: Molecules. 2026 Sep 1;31(17):3078. doi: 10.3390/molecules31173078 (PMC13567080; doi:10.3390/molecules31173078)
Supplement: Supplementary file 1 [file molecules-31-03078-s001.zip › molecules-4519180-supplementary.pdf]

S1. Computational details

Density functional theory (DFT) calculations were performed using the Vienna Ab initio Simulation Package (VASP) [55,56]. The exchange–correlation interactions were described within the generalized gradient approximation (GGA) using the Perdew–Burke–Ernzerhof (PBE) functional [57]. The projector augmented wave (PAW) method was employed to treat the electron–ion interactions [58]. A plane-wave cutoff energy of 400 eV was used. Due to the large supercell, the Brillouin zone was sampled using a 2 × 2 × 1 Monkhorst–Pack k-point grid. Structural optimizations were carried out until the residual force on each atom was below 0.02 eV $Å^{-1}$, with an energy convergence criterion of 1.0 × 10^{-4} eV. Van der Waals interactions were included using the DFT-D3 method [59].

A $Ti_3C_2T_x$ slab model with surface terminations consistent with experimentally obtained MXene was constructed, and a vacuum region of more than 15 Å was introduced along the surface normal to avoid interactions between periodic images. CNT fragments and short PEO chains were introduced to simulate the composite interface.

The adsorption energy (E ads) was calculated as:

$$E_{ads} = E_{surface+LiPSs} - E_{surface} - E_{LiPSs}$$

where $E_{surface+LiPSs}$, $E_{surface}$, and E_{LiPSs} represent the total energies of the adsorption system, the clean surface, and the isolated LiPSs species, respectively.[60].

S2. Adsorption energy analysis

The adsorption energies of lithium polysulfide species (Li_2S_2, Li_2S_4, Li_2S_6, and Li_2S_8) and S_8 on the MX/CNT/PEO interface were calculated to evaluate the interfacial binding capability. As summarized in Table S1, the MX/CNT/PEO interface exhibits strong adsorption toward lithium polysulfides, with adsorption energies ranging from −5.534 to −8.331 eV.

Notably, the adsorption strength increases with the chain length of polysulfides, with Li_2S_8 showing the strongest interaction. This trend suggests stronger interfacial affinity of long-chain LiPSs toward the MX/CNT/PEO interface. In contrast, the

relatively weaker adsorption of S_8 suggests preferential stabilization of soluble polysulfides rather than solid sulfur, which is consistent with the reduced polysulfide diffusion observed experimentally.

Table S1. Adsorption energies of LiPS species on the MX/CNT/PEO interface

Species	Adsorption energy (eV)
Li_2S_2	−7.230
Li_2S_4	−5.534
Li_2S_6	−7.359
Li_2S_8	−8.331
S_8	−5.693

S3. Additional structural characterization

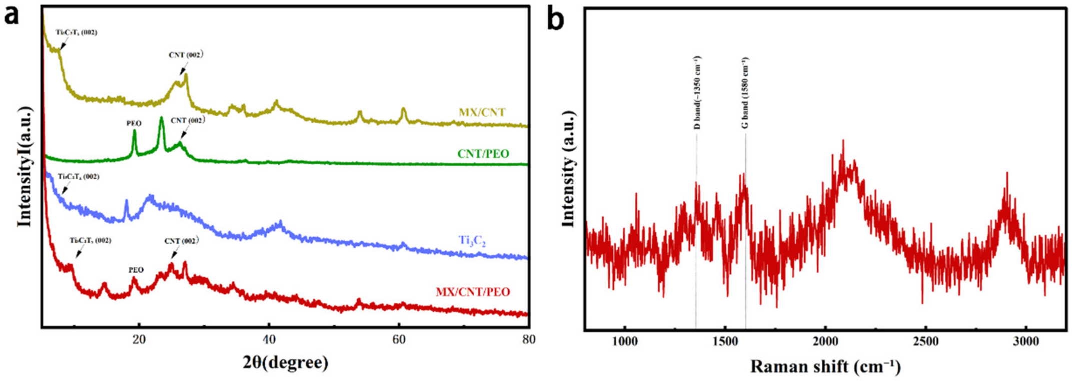

Figure S1. Additional structural characterization of the MX/CNT/PEO composite. (a) XRD patterns of MX/CNT/PEO, $Ti_3C_2T_x$ MXene, CNT/PEO, and MX/CNT samples. The low-angle peak is assigned to the (002) reflection of $Ti_3C_2T_x$ MXene. The broad diffraction peak around 25–26° corresponds to the graphitic (002) plane of CNTs, while the reflections around 19° and 23° are associated with crystalline PEO domains. (b) Raman spectrum of the MX/CNT/PEO composite. The D and G bands at approximately 1350 and 1580 cm^{-1} are associated with defective/disordered carbon and sp^2-hybridized graphitic carbon, respectively.

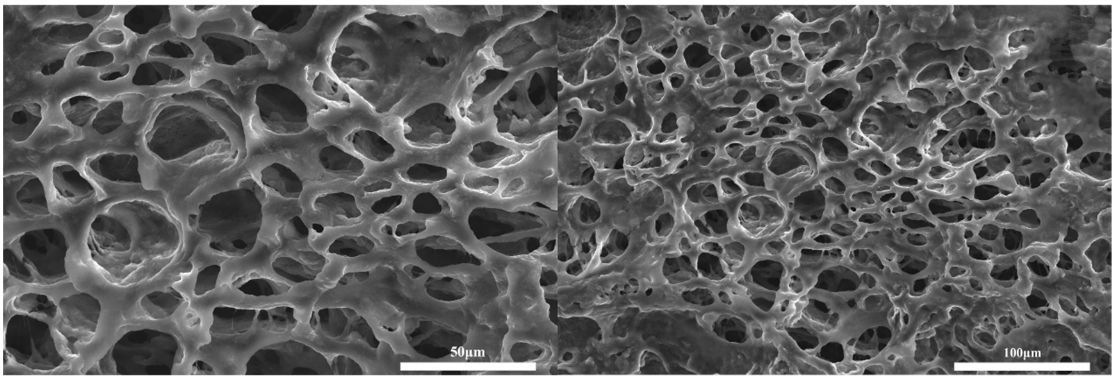

Figure S2. Additional SEM images of the MX/CNT/PEO composite at different magnifications, revealing a well-interconnected three-dimensional porous architecture. The uniform pore distribution and continuous framework facilitate electrolyte penetration and provide continuous pathways for ion transport, which are expected to promote efficient interfacial ion transport within the separator.

S4. XPS analysis (supplementary)

Table S2. Binding energies and assignments of XPS peaks for the MX/CNT/PEO composite.

Element	Peak (eV)	Assignment
Ti $2p_{3/2}$	454.6	Ti–C
Ti $2p_{3/2}$	458.5	Ti–O
C 1s	284.6	C–C/C=C
C 1s	286.2	C–O
C 1s	288.5	O–C=O
O 1s	530.1	Ti–O
O 1s	531.5	C=O / O–C=O
O 1s	532.8	C–O

References

[1] G. Kresse, J. Hafner, Phys. Rev. B 47 (1993) 558–561.

[2] G. Kresse, J. Furthmüller, Phys. Rev. B 54 (1996) 11169–11186.

[3] J.P. Perdew, K. Burke, M. Ernzerhof, Phys. Rev. Lett. 77 (1996) 3865–3868.

[4] G. Kresse, D. Joubert, Phys. Rev. B 59 (1999) 1758–1775.

[5] S. Grimme, J. Comput. Chem. 27 (2006) 1787–1799.

[6] K. Momma, F. Izumi, J. Appl. Crystallogr. 44 (2011) 1272–1276.
